# Data set for the proteomic inventory and quantitative analysis of chicken eggshell matrix proteins during the primary events of eggshell mineralization and the active growth phase of calcification

**DOI:** 10.1016/j.dib.2015.06.019

**Published:** 2015-07-07

**Authors:** Pauline Marie, Valérie Labas, Aurélien Brionne, Grégoire Harichaux, Christelle Hennequet-Antier, Alejandro B. Rodriguez-Navarro, Yves Nys, Joël Gautron

**Affiliations:** a INRA, UR83 Recherches avicoles, Fonction et Régulation des protéines de l׳œuf, F-37380 Nouzilly, France; b INRA, UMR INRA85, UMR CNRS 7247; Université de Tours; IFCE, Physiologie de la Reproduction et des Comportements, Plate-forme d׳Analyse Intégrative des Biomolécules, Laboratoire de Spectrométrie de Masse, F-37380 Nouzilly, France; c Departmento de Mineralogia y Petrologia, Universidad de Granada, Campus Fuente Nueva s/n, 18002 Granada, Spain

**Keywords:** Chicken, Eggshell, Biomineralisation, Quantitative proteomics, Eggshell matrix

## Abstract

Chicken eggshell is a biomineral composed of 95% calcite calcium carbonate mineral and of 3.5% organic matrix proteins. The assembly of mineral and its structural organization is controlled by its organic matrix. In a recent study [1], we have used quantitative proteomic, bioinformatic and functional analyses to explore the distribution of 216 eggshell matrix proteins at four key stages of shell mineralization defined as: (1) widespread deposition of amorphous calcium carbonate (ACC), (2) ACC transformation into crystalline calcite aggregates, (3) formation of larger calcite crystal units and (4) rapid growth of calcite as columnar structure with preferential crystal orientation. The current article detailed the quantitative analysis performed at the four stages of shell mineralization to determine the proteins which are the most abundant. Additionally, we reported the enriched GO terms and described the presence of 35 antimicrobial proteins equally distributed at all stages to keep the egg free of bacteria and of 81 proteins, the function of which could not be ascribed.

**Specifications table**

**Table:** 

Subject area	*Biology*
More specific subject area	List and putative functions of eggshell matrix proteins present at initial and mid phases of eggshell formation.
Type of data	Raw and processed/analyzed mass spectrometry data obtained by nanoliquid chromatography combined to high resolution tandem mass spectrometry,.xls tables with identified/validated and quantified proteins tables with integrative and functional analysis of protein sequences.
How data was acquired	LC–MS/MS using a LTQ Orbitrap Velos mass spectrometer.
Data format	Raw data: raw.mzml and Processed and analyzed data using Mascot Search engine:.dat.
	Analyzed: Further assembled sequences using Clustal Omega multi-alignment algorithm and BLAST+suite. GO terms extracted from sequences and enrichment determination.
Experimental factors	Stage of eggshell formation.
Experimental features	Eggshell matrix samples were collected at four stages of eggshell mineralization and digested in gel using trypsin. Resulting peptides were analyzed by LC–MS/MS and further treated using data mining and bioinformatic analysis.
Data source location	Nouzilly, France, INRA Centre Val de Loire.
Data accessibility	Data have been deposited into the ProteomeXchange Consortium [2] via the PRIDE partner repository with the dataset identifier PXD001450.

*Value of the data* [describe in 3–5 bulleted points why this data is of value to the scientific community]

• Proteomic analysis of 216 chicken eggshell matrix proteins.
• Gene Ontology terms enrichments investigating potential functions of eggshell matrix proteins.
• Quantitative data on protein abundances according to stages of eggshell formation.
• Annotation on quantified eggshell matrix proteins.

## 1 Collection of eggshell matrix samples and preparation for MS analyses

Eggs were collected on brown-laying hens at 5, 6, 7 and 16 h after previous oviposition as described in [1]. These time intervals correspond respectively to the primary events of mineralization with widespread deposition of amorphous calcium carbonate (ACC), ACC transformation into crystalline calcite aggregates, formation of larger calcite crystal units and rapid growth of calcite and development of a columnar structure with preferential crystal orientation [3]. Eggs were broken and forming eggshells were washed with water, air dried and stored at −20 °C until protein extraction. Eggshell matrix proteins were extracted as described in [1].

A total of 24 individual eggshell protein extracts were used. Six samples collected at the same time point were pooled in equal amounts for each time (5 h p.o., 6 h p.o., 7 h p.o. and 16 h p.o.). The four pooled samples (51 µg of proteins/sample) were fractionated on a 4–20% SDS-PAGE gel (8.3 cm×7.3 cm×1.5 mm). Proteins were stained with Coomassie blue and the entire SDS-PAGE lanes were sectioned into 15 bands for each individual pooled sample. Excised proteins were in-gel digested with bovine trypsin (Roche Diagnostics GmbH, Mannhiem, Germany) as previously described [4] and analyzed by nanoscale liquid chromatography-tandem mass spectrometry (nanoLC–MS/MS).

## 2 Nano LC MS/MS analyses

All experiments were performed on a linear ion trap Fourier Transform Mass Spectrometer (FT-MS) LTQ Orbitrap Velos (Thermo Fisher Scientific, Bremen, Germany) coupled to an Ultimate^®^ 3000 RSLC Ultra-High Pressure Liquid Chromatographer (Dionex, Amsterdam, The Netherlands) as previously described in [1,4]. Raw data files were converted to MGF as previously described [4]. The identification of proteins was established using MASCOT search engine (v 2.3, Matrix Science, London, UK). The peptide and fragment masses obtained were matched automatically against the chordata section of nr NCBI database (2132453 sequences, downloaded on 2013/05/13). Enzyme specificity was set to trypsin with two missed cleavages using carbamidomethylcysteine, oxidation of methionine and N-terminal protein acetylation as variable modifications. The tolerance of the ions was set to 5 ppm for parent and 0.8 Da for fragment ion matches. Mascot results obtained from the target and decoy database were incorporated in Scaffold 3 software (version 3.4, Proteome Software, Portland, USA). Peptide identifications were accepted if they could be established at greater than 95.0% probability as specified by the Peptide Prophet algorithm [5]. Peptides were considered distinct if they differed in sequence. Protein identifications were accepted if they could be established at greater than 95.0% probability as specified by the Protein Prophet algorithm [6] and contained at least one sequence-unique peptide for global inventory and at least two sequence-unique peptides for quantitative analysis. A false discovery rate was calculated as <1% at the peptide or protein level. The abundance of identified proteins was estimated by calculating the emPAI using Scaffold 4 Q+ software (version 4.2, Proteome Software, Portland, USA). Additionally, label-free quantitative proteomic analyses based on spectral counting method, Scaffold 3 Q+ software (version 3.4, Proteome Software, Portland, USA) was used to quantify the proteins at the four different stages of calcification. All proteins with greater than two sequence-unique peptides, identified in database with high confidence were considered for protein quantification. To eliminate quantitative ambiguity within protein groups, we ignored all the spectra matching any peptide which is shared between proteins. Thereby, quantification performed with normalized spectral counts was carried out on distinct proteins.

The mass spectrometry proteomic data have been deposited into the ProteomeXchange Consortium [2] via the PRIDE partner repository with the dataset identifier PXD001450.

## 3 Data set analysis of avian eggshell matrix proteins

A total of 261 proteins were identified using nr NCBI database. Keratins and bovine trypsin were eliminated from the list as they appeared to be contaminants or resulting from the digestion process. Protein sequences were aligned to eliminate all redundancies. Protein groups were determined using Clustal Omega multi-alignment algorithm [7]. Sequences were blasted against nr NCBI database limited to Gallus gallus taxon using the blastp program (BLAST+suite) [8]. This was performed using R language (http://cran.r-project.org).

A total of 216 non-redundant eggshell matrix protein sequences were identified. The resulting file (Supplementary Table 1) is made of EntrezGene Ids, GI numbers, protein symbols, short descriptions, information on their previous identification in the shell and in the uterine fluid, mean quantitative values and emPAI values at each time point. We compared our list with the previously published proteomes of eggshell matrix proteins [9–15]. Out of the 216 proteins, 24 proteins are novel compared to previous studies and are highlighted (Supplementary Table 1).

In order to determine the potential functions of the 216 eggshell matrix proteins, 3231 GO terms were extracted from the protein sequences using GORetriever (http://agbase.msstate.edu/). CateGOrizer (http://www.animalgenome.org/tools/catego/) was then used to group the GO terms in 91 different parent term categories using a GO slim2 method. Finally, GO terms enrichments were determined using Gene set enrichment tools from Genomatix suite (www.genomatix.de). When considering GO terms associated to Molecular Function (MF) and Biological Process (BP), a total of 71 GO terms were found to be significantly enriched (*p*-values ranges from 9.92·10^−3^ to 2.96·10^−7^). They were grouped in 18 main categories (Table 1). Groups of importance were composed of eggshell matrix proteins involved in binding and transport activity (41 proteins) and of 27 proteins related to the biology of development. Additionally, we also reported proteins involved in response to external stimulus (13 proteins), in the regulation of biological quality (15 proteins), exhibiting enzyme regulator activity (13 proteins). Also were present proteins related to protein post-translational folding (9 proteins), homeostasis maintenance (9 proteins), multi-organism process (8 proteins), nutrition and digestion (7 proteins), the extracellular organization (4 proteins), the cell–cell adhesion (5 proteins), the oxidoreductase activity (3 proteins), the coagulation process (2 proteins), the regulation of protein phosphorylation (2 proteins), the glycerol ether metabolic process (2 proteins) and the shell calcification (2 proteins).

## 4 Quantitative dataset of avian eggshell matrix proteins

Two different methods were applied to discern the relative abundance of the proteins at the different stages of eggshell formation, firstly the emPAI was calculated for all proteins at each individual stage and secondly, GeLC–MS/MS analyses combined with label free spectral counting method were carried out to determine quantitative values at the four stages of mineralization for the 216 unique proteins.

In the first approach, emPAI from the proteins was calculated amongst the proteins revealed at a particular stage of shell formation at 5, 6, 7 and 16 h p.o. The aim was to classify the relative abundance of the proteins within one individual stage and to determine those which were the most abundant ones. EmPAI values were reported for the 216 eggshell matrix proteins at each stage, a numerical value different from zero being introduced when the protein was present (Supplementary Table 1). EmPAI values were also used to determine the number of proteins present at a particular stage (Supplementary Table 2). The numbers of proteins showing an emPAI different from zero were 91, 132, 178 and 184 at 5, 6, 7 and 16 h p.o., respectively.

In the second approach, GeLC–MS/MS combined with label free quantitative analyses based on a spectral counting method were used to compare the abundances of the different proteins between the four stages of eggshell formation (Supplementary Tables 1 and 2). One way ANOVA was performed on quantitative values for protein abundance in order to reveal proteins which were at a different concentration between at least one stage relative to another one. Differences were considered to be statistically significant for *p*-value<0.01. Amongst the 216 proteins, 175 showed differential abundance according to the four stages of shell calcification. The mean abundance per stage of shell calcification was standardized for each protein on mean values per stage and was calculated to perform hierarchical clustering analysis. A total of ten clusters were highlighted and detailed in [1]. They were grouped in five main protein profiles associated with the different events which occur during eggshell calcification [1,3]: overabundance during the primary events of widespread deposition of amorphous calcium carbonate, totaling 27 proteins (5–6 h p.o.), overabundance during transformation of ACC into crystalline calcite aggregates, totaling 13 proteins (6 h p.o.), overabundance during the formation of larger calcite crystal units, totaling 47 proteins (6–7 h p.o.), overabundance during rapid growth of calcite and development of a columnar structure with preferential orientation of calcite, totaling 70 proteins (16 h p.o.). The last group corresponded to overabundance throughout the three late stages of calcification (6–7 to 16 h p.o.), totalising 18 proteins.

## 5 Supplementary functional annotations of avian eggshell matrix proteins

Potential functions of proteins were determined according to the literature, data annotations and functional domain database and highlighted 77 proteins with potential functions related to a mineralization process [1].

Beside these proteins, we identified 35 proteins with potential antimicrobial functions present at various stages of mineralization (Supplementary Table 3). These antimicrobial proteins were present a quite equally number throughout the different stages of shell formation.

The most abundant protein in the shell matrix is *lysozyme*, which is known to hydrolyze 1–4 beta linkages between N-acetyl muramic acid and N-acetyl-d-glucosamine residues in peptidoglycans of Gram-positive bacteria [16]. Another notable proteins is *OVAX*, which exhibits antimicrobial properties against *Listeria monocytogenes* and *Salmonella* enteritidis [17]. *Avian β defensin 11*, belonging to avian β defensin family, is a cationic peptides with three standed β sheet structure connected with β hairpin loop that protect against Gram-positive and Gram-negative bacteria [18]. *Ovocledin-17*, a C-type lectin protein which exhibits antimicrobial properties against *Bacillus subtilis*, *Staphylococcus aureus* and *Pseudomonas aeruginosa* is also a major eggshell protein [19].

Another group is constituted of antimicrobial proteins by depriving bacteria of essential nutrients. *Ovotransferrin*, *vitellogenin***-***1 and 2* and *MFI2* are antimicrobial by their capacity to chelate iron ions, essential for bacterial growth [20]. *GC*, a group-specific component and *riboflavin protein* bind vitamins which are depriving bacteria from vitamin D.

We report *ovocalyxin-36*, *BPIFCB* and *ovoglobulinG2 type AA*, which belong to the LBP/BPI/PLUNC family known to bind to the lipid A portion of lipopolysaccharide cell wall in Gram-negative bacteria leading to the death of bacteria [21].

The study also revealed protease inhibitors which can be potentially antimicrobial by their ability to inhibit proteases secreted by some bacteria. *Ovoinhibitor*, *ovomucoid***,** *SPARC***,** *follistatin*, *follistatin-like 1* and *IGFBP7* exhibit Kazal like protease inhibitor domains. Moreover, ovoinhibitor was showed to inhibit *Bacillus thuringiensis* growth [22]. *Ovocalyxin-32* presents homology with latexin, a carboxypeptidase inhibitor, and has been shown to inhibit *B. subtilis* growth [23]. *SERPIND1*, *SERPINF2*, *SERPINE2*, *SERPING1* and *SERPINI1* belong to SERPIN family, a known family of serine protease inhibitors [16]. *Ovocalyxin-25* contains two inhibitor protease domains, a WAP and a Kunitz-like domain [24]. *Cystatin C* is a cysteine protease inhibitor, and *ovostatin* is known to inhibit all four classes of proteases [16].

Finally our study revealed several fragment of immunoglobulin (*Ig-light-2*, *Ig-gamma-heavy*, *Ig-lambda-1*, *Ig-lambda-2*, *Ig-light-1*, *Ig-mu-heavy* and *Ig-alpha-heavy*).

Finally, out of the 175 eggshell matrix proteins showing significant variation of abundance according to the four stages of shell calcification, 81 could not be ascribed to any of these functional groups and were classified as “other or unknown role” (Supplementary Table 4).

## Conflict of interest

Authors claim no conflict of interest.

## Figures and Tables

**Table 1:** GO terms significantly enriched in the 216 identified eggshell matrix proteins.

**Descriptions**	**Number of proteins**	**Symbols**
Response to external stimulus	13	AVBD11, HSPA5, RBP4, FN1, LCN8, ALB, OC-17, CTSD, HIST1H2B7, LYZ, APOA1, TTR, LY86
Extracellular organization	4	ANXA2, LOXL2, VMO1, COL2A1
Nutrition and digestion	7	HSPA5, ALB, CTSD, RBP4, APOA1, VTG1, VTG2
Oocyte formation	3	RBP4, VMO1, TTR
Protein post translational folding	9	PDIA3, PPIB, QSOX1, P4HB, HSPA5, DNAJC3, HSP90B1, HSP90AA1, DNAJB6
Homeostasis maintenance	9	PDIA3, RBP4, CALB1, QSOX1, LCN8, COL2A1, P4HB, APOA1, CALM
Development	27	HSPA5, SPP1, RBP4, SEMA3C, DKK3, ACTA1, ANXA2, LOXL2, CALB1, YWHAE, FN1, ATP5B, LCN8, CDH2, TSKU, OC-17, GSN, DNAJB6, COCH, COL2A1, FST, SDF4, OC-116, APOA1, CNTN1, CA2, TTR
Enzyme regulator activity	13	HSP90AA1, ANXA2, OVM, OVST, OVAY, SERPINI1, DNAJB6, APOA1, OIH, OVAX, TIMP2, CST3, CALM
Binding and transport activity	41	DNAJC3, DNAJB6, CALB1, APOA1, GPC1, CNTN1, ALB, LCN8, HSPA5, SPP1, RBP4, HSP90B1, PTN, HSP90AA1, ANXA2, LOXL2, OVM, YWHAE, RDX, ANXA5, CDH2, GSN, COL2A1, FST, YWHAZ, HIST1H2B7, OIH, LYZ, HBG2, CDH1, TTR, CALM, FBLN1, SDF4, SPARC, OC-17, PLOD1, HBAA, HBAD, VTG1, VTG2
Oxidoreductase activity	3	PDIA3, QSOX1, P4HB
Coagulation process	2	ANXA2, ANXA5
Cell–cell adhesion	5	CDH2, DNAJB6, COL2A1, APOA1, CDH1
Cartilage metabolism	2	LOXL2, LCN8, CDH2, COL2A1
Glycerol ether metabolic process	2	PDIA3, P4HB
Regulation of biological quality	15	PDIA3, RBP4, ANXA2, ALB, CALB1, FN1, QSOX1, RDX, LCN8, GSN, COCH, COL2A1, P4HB, APOA1, CALM
Multi-organism process	8	RBP4, ALB, AVBD11, LCN8, OC-17, HIST1H2B7, LYZ, TTR
Shell calcification	2	OC-17, OC-116
Regulation of protein Dephosphorylation	2	YWHAE, CALM

## Appendix A Supplementary materials

Supplementary data. Supplementary Table 1: Excel file describing the 216 non redundant eggshell matrix proteins. Newly identified proteins are underlined. Supplementary Table 2: Excel file describing the emPAI and quantitative values at each individual stage (5, 6, 7 and 16 hours post ovulation). Supplementary Table 3: Excel file describing eggshell matrix proteins potentially antimicrobials. Supplementary Table 4: Excel file describing eggshell matrix proteins which could not be ascribed to functional role related to mineralization or antimicrobial properties.
